# Miscellany

**Published:** 1902-12

**Authors:** 


### Miscellany.

Collapsed while extracting under Chloroform. — On Saturday, the 5th inst., about 7.30 p.m., a woman and her husband called at my office. The woman was suffering severely from toothache, and had been for some days. On examining the mouth I found the whole upper denture more or less decayed, and some eight of the lower ones also, the pain being caused principally by a lower bicuspid and third molar. I advised the removal of the aching ones, and let the others remain for the present. But the lady, having suffered severely during extraction before, stated that she wanted chloroform, and would have all the decayed ones out at the one time. I sent her to her physician for examination, saying that if she was a fit subject I was satisfied. She was pro-

---

nounced a good subject by her physician, and the chloroform was administered by him at 11.30 A.M. the following day. She took the anæsthetic well, everything being apparently satisfactory, and in about ten minutes was completely under the anæsthetic. I started to operate, and had removed thirteen teeth when the collapse occurred, and breathing ceased. Efforts were at once begun to revive her, artificial respiration, hypodermic injections of strychnine and nitroglycerin, also brandy, etc., being used, but without any result whatever, and after about an hour's work abandoned, as the case was hopeless. A coroner's inquest was held next day, the verdict being that the deceased came to her death during the administration of chloroform for the extraction of teeth, through paralysis of the respiratory centre, and that no blame was to be attached to any parties connected with operation.—W. J. HILL, Ontario, *Dominion Dental Journal.*

